# SpaGraphCCI: Spatial cell–cell communication inference through GAT‐based co‐convolutional feature integration

**DOI:** 10.1049/syb2.70000

**Published:** 2025-01-23

**Authors:** Han Zhang, Ting Cui, Xiaoqiang Xu, Guangyu Sui, Qiaoli Fang, Guanghao Yang, Yizhen Gong, Sanqiao Yang, Yufei Lv, Desi Shang

**Affiliations:** ^1^ School of Computer University of South China Hengyang Hunan China; ^2^ The First Affiliated Hospital Cardiovascular Lab of Big Data and Imaging Artificial Intelligence Hengyang Medical School University of South China Hengyang Hunan China; ^3^ Hunan Provincial Key Laboratory of Multi‐omics and Artificial Intelligence of Cardiovascular Diseases University of South China Hengyang Hunan China; ^4^ Department of Biochemistry and Molecular Biology School of Basic Medical Sciences Hengyang Medical School University of South China Hengyang Hunan China; ^5^ The First Affiliated Hospital Institute of Cardiovascular Disease Hengyang Medical School University of South China Hengyang Hunan China; ^6^ Department of Cardiology The First Affiliated Hospital Hengyang Medical School University of South China Hengyang China; ^7^ Chinese Medicine Hospital of Daqing Daqing China; ^8^ Department of Anesthesiology The First Affiliated Hospital Hengyang Medical School University of South China Hengyang Hunan China; ^9^ Department of Human Anatomy Hengyang Medical School University of South China Hengyang Hunan China; ^10^ Department of Cell Biology and Genetics School of Basic Medical Sciences Hengyang Medical School University of South China Hengyang Hunan China

**Keywords:** bioinformatics, feature extraction, graphs, learning (artificial intelligence)

## Abstract

Spatially resolved transcriptomics technologies potentially provide the extra spatial position information and tissue image to better infer spatial cell–cell interactions (CCIs) in processes such as tissue homeostasis, development, and disease progression. However, methods for effectively integrating spatial multimodal data to infer CCIs are still lacking. Here, the authors propose a deep learning method for integrating features through co‐convolution, called SpaGraphCCI, to effectively integrate data from different modalities of SRT by projecting gene expression and image feature into a low‐dimensional space. SpaGraphCCI can achieve significant performance on datasets from multiple platforms including single‐cell resolution datasets (AUC reaches 0.860–0.907) and spot resolution datasets (AUC ranges from 0.880 to 0.965). SpaGraphCCI shows better performance by comparing with the existing deep learning‐based spatial cell communication inference methods. SpaGraphCCI is robust to high noise and can effectively improve the inference of CCIs. We test on a human breast cancer dataset and show that SpaGraphCCI can not only identify proximal cell communication but also infer new distal interactions. In summary, SpaGraphCCI provides a practical tool that enables researchers to decipher spatially resolved cell–cell communication based on spatial transcriptome data.

## Graphical abstract

Spatially resolved transcriptomics (SRT) technologies potentially provide the extra spatial position information and tissue image to better infer spatial cell–cell interactions (CCIs) in processes such as tissue homeostasis, development, and disease progression. However, methods for effectively integrating spatial multimodal data to infer CCIs are still lacking. Here, we propose a deep learning method for integrating features through co‐convolution, called SpaGraphCCI, to effectively integrate data from different modalities of SRT by projecting gene expression and image feature into a low‐dimensional space.

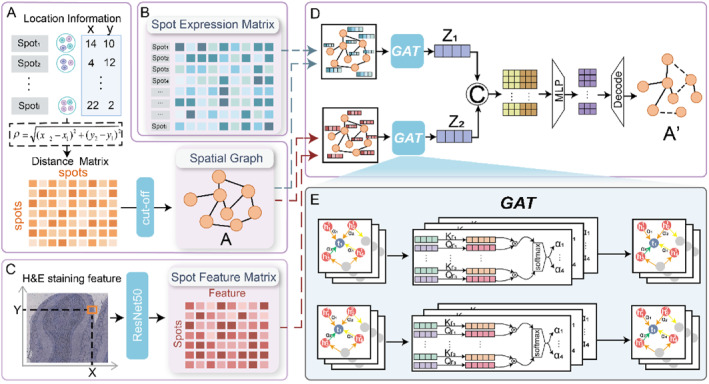

## 1 INTRODUCTION

Spatial transcriptomics (ST) provides valuable spatial information about cells or spots composed of multiple or partial cells [1]. These techniques facilitate the measurement of spatial gene expression in two‐dimensional (2D) or three‐dimensional (3D) tissue samples with varying degrees of cellular resolution [2]. At present, spatially resolved transcriptomics (SRT) sequencing technology has catalysed important advancements, such as in situ sequencing methods FISSEQ [3] and STAR‐map [4]; fluorescence in situ hybridisation (FISH)‐based imaging methods such as MERFISH [5], seqFISH [6], SPOTs [7] and osmFISH [8]; spatial barcode techniques such as Slide‐seq [9], HDST [10], 10X Visium [11], and DBiT‐seq [12], as well as laser capture microdissection combined with flow cytometry methods such as GEO‐seq [13] and TSCS [14]. The coverage of these transcriptome profiles ranges from tens to thousand genes due to different experimental designs.

The emergence of SRT significantly enhances the accuracy and reliability of spatial CCC inference in biology and biomedicine [15]. Cell–cell interactions (CCIs) are the basis of multicellular life and are necessary for biological functions. Cell–cell interactions produce various molecules and membrane structures that activate signalling pathways in other cells, regulating gene expression and driving cellular functions. Recently, several approaches have emerged to explain the underlying mechanisms of CCI within a spatial context, including CellPhoneDB v3 (CPDB3) [16], stLearn [17], SVCA [18], MISTy [19], NCEM [20], Giotto [21], SpaOTsc [22], and COMMOT [23]. Despite the popularity of these methods and their significant contribution to the field of cell–cell communication, many of these tools only infer interactions between cell types and neglect the analysis of CCI between paired individual cells, thus losing the single‐cell resolution. CellPhoneDB v3 (CPDB3) restricts interactions between cell types located in the same microenvironment as determined by spatial information. SpaOTsc performs structured optimal transport mapping between scRNA‐seq and ST data, assigns spatial locations to cells, and uses distance between cells as transport costs to infer ligand–receptor signalling networks that regulate spatial constraints. And these methods rely on the co‐expression of ligand–receptor gene pairs in signal‐sending and signal‐receiving to detect CCIs. Due to the high sparsity and noisy nature of spatial transcriptome data, the coverage of ligand and receptor genes is both limited and uneven, making it challenging to directly apply traditional methods based on known ligand–receptor pairs. Furthermore, the incompleteness of this prior knowledge, particularly for specific cell types or tissues, can result in the omission of essential information, leading to an incomplete identification of cell–cell communication relationships.

In addition, some deep learning‐based methods to infer CCIs from spatially resolved transcriptome data have been developed. For example, DeepLinc [24] implements an autoencoder to infer CCI networks, considering both expression data and cell graph inputs, and reconstructs the communication between cells by decoding the latent feature representation of cells. Another method, Clarify [25], uses encoders to generate gene‐level and cell‐level latent features separately and infers CCI by integrating gene‐level and cell‐level latent features. However, due to the limitations of current sequencing technologies in sequencing depth and coverage, the obtained data are often affected by a large amount of noise and dropout. The identification of CCI of spatial transcriptomes based on expression data obtained by sequencing alone might affect the accuracy of inference due to high data noise [26].

Therefore, we propose SpaGraphCCI, which learns feature information from spatial expression profiles, cell networks, and pathology images through graph attention. SpaGraphCCI integrates these multimodal features and projects them into a low‐dimensional space to compensate for high noise and dropout during sequencing. By decoding the integrated embedded representations, SpaGraphCCI can infer spatially resolved CCIs. SpaGraphCCI is able to efficiently infer spatial CCIs, and performance evaluation shows that SpaGraphCCI exhibits significant advantages over other methods. We applied SpaGraphCCI to HDST and 10X Visium datasets to reveal the cell communication mechanisms in the spatial structure diseased tissues. Overall, the results show that SpaGraphCCI helps to resolve spatial CCIs in ST data at both single‐cell resolution and spot resolution, providing valuable insights into understanding spatial cellular dynamics in tissues.

## 2 DATASETS

### 2.1 Dataset description

The SpaGraphCCI analysis used six published datasets covering both single‐cell resolution and spot‐based spatial transcriptomic data [27]. These datasets include single‐cell resolution datasets from the HDST platform (mouse olfactory bulb and human breast cancer datasets) and spot resolution datasets from the 10X Visium platform (human small intestine, human colon, mouse liver, and human dorsolateral prefrontal cortex datasets).

The HDST dataset provides high‐throughput transcriptome profiles of approximately 10,000 genes, of which the mouse olfactory bulb and human breast cancer datasets were downloaded from the Broad Institute's data portal (https://portals.broadinstitute.org/single_cell/study/SCP420 [10]). For the human breast cancer dataset, we selected the CN21_E2 region (x:8464–9664, y:5000–6500) containing 1765 cells, and for the mouse olfactory bulb dataset, we selected the CN13_D2 region (x:8447–11,447, y:5447.5–6447.5) containing 1981 cells.

For the spot resolution‐based datasets, we obtained from the SODB [28] data platform in which the human small intestine dataset [29] contains 346 spots covering 13,231 genes after processing, and the human colon dataset [29] contains 644 spots covering 13,601 genes. In addition, the mouse liver [30] dataset contains 590 and 673 spots, respectively, and the human dorsolateral prefrontal cortex [31] dataset from SpatialRef [32] contains 4226 spots. These diverse datasets provide a solid foundation for SpaGraphCCI to analyse the spatial communication patterns of different tissues and cell types.

### 2.2 Model input

Spatial transcriptomics sequencing technology provides gene expression profiles accompanied by additional spatial location information and tissue image information, which provide a unique perspective on intercellular communication in the tissue microenvironment. SpaGraphCCI makes full use of these additional tissue data to enhance gene expression associations at adjacent points to more accurately infer spatial cell communication.

For spatial transcriptome (ST) data containing morphological information, SpaGraphCCI segments the tissue image according to the coordinates of each point (such as H&E stained sections), thereby obtaining a local image of each point. These local images capture microscopic tissue morphological structure information, which can reflect the spatial layout of cells and their interactions in the tissue. Next, the pre‐trained ResNet50 [33] is used to extract features from these local images to obtain rich image features.

(1) $$\text{Region}\left(X,Y\right)=I\left[X-\frac{W}{2}:X+\frac{W}{2},X-\frac{H}{2}:X+\frac{H}{2}\right]$$

where I represents the input tissue image; X,Y denote the centre coordinates of the segmentation region; and W,H denote the width and height of the local image to be extracted, respectively.

For gene expression data processing, we used a gene filtering step to reduce the interference of noise on the analysis results. Specifically, we filtered out genes that were expressed in fewer than 10 spots. The aim of this strategy is to exclude those rarely expressed or low‐abundance genes, which often fail to provide meaningful biological information and may instead introduce noise, affecting the accuracy and stability of the model [34, 35]. This preprocessing step helps to reduce errors and improve the quality of spatial cell communication network inference.

SpaGraphCCI learns transcriptomic features related to CCIs by using neighbouring cells with direct contact as a positive sample set. It is reasonable to assume that most cells are capable of making direct contact with five or more other cells in solid tissues. In order to optimise the model, it is crucial to reduce the potential false positive (FP) samples in the positive sample set during training. Therefore, for each cell, we selected only the five nearest neighbours to define the direct contact relationship. We believe that this is an appropriate balancing choice that provides a sufficient number of directly contacted cells to support effective training of the SpaGraphCCI model. At the same time, it effectively reduces the interference of false positive samples in the positive training set. Through this strategy, we ensured that sufficient and accurate information about CCIs was obtained during the model learning process while maintaining the high quality and reliability of the dataset.

## 3 METHOD

### 3.1 Overall architecture

**FIGURE 1:**
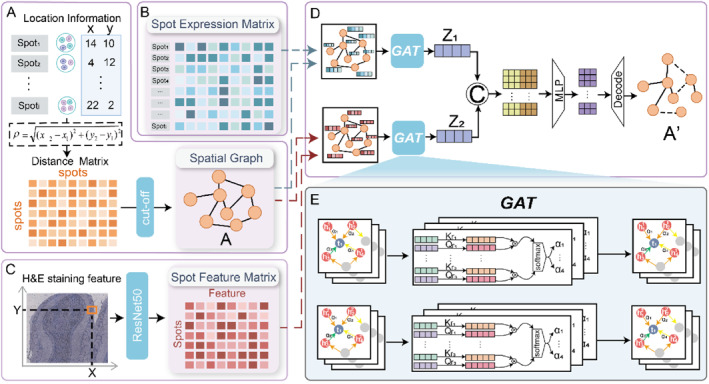
Overview of SpaGraphCCI. (a) Construct the spatial graph according to the coordinates. (b) Spot expression matrix. (c) Resnet50 was used to extract image features. (d) SpaGraphCCI uses a dual‐stream graph attention network to predict cell‐to‐cell communication. (e) Details of the GAT module.

The following Figure 1 illustrates our proposed SpaGraphCCI architecture for the task of identifying spatial cellular communication.

(i) A spatial network was constructed based on the coordinate information as shown in Figure 1a, and the gene expression counting matrix as shown in Figure 1b and the feature matrix extracted from pathological images as shown in Figure 1c were used as input to the model;
(ii) In this model, the graph attention network (GAT) with the multi‐head attention mechanism was employed. We designed two separately trained GATs, one processing the spatial network with node features as gene expression and the other processing the spatial network with node features as image feature (Figure 1d). Learning the gene expression features and image information of the spatial network through independent GAT structures and generating the corresponding embedded feature representation help to improve the prediction ability of the model for complex CCIs.
(iii) To further improve the ability of the model to process multimodal data, the embedded features learnt by the two GAT are fused. Specifically, we employ a multi‐layer perceptron (MLP) to reduce the dimensionality of the two embedded feature representations to ensure that the features of different modalities can be represented in a more compact and effective way. The fusion features after dimension reduction can capture the information of image and gene expression data at the same time, and the reconstructed spatial cell communication network is obtained by the decoder module.
(iv) Finally, the decoder module was used to generate the reconstructed cell communication network, which revealed the communication mechanism between cells in the spatial structure.

### 3.2 Dual‐stream graph attention network

At the heart of the GAT network is the multi‐head attention mechanism, as shown in Figure 1e. GAT is able to effectively model cellular relationships on a graph‐based basis, which is essential for inferring CCI. A key advantage of GAT is its attention mechanism, which dynamically assigns different attention scores based on the connectivity characteristics between nodes. This allows the model to focus on the most relevant relationships. The input feature matrix is passed to multiple parallel attention heads. Specifically, the graph attention mechanism dynamically captures the relative importance or correlation between nodes by assigning an attention score to each pair of nodes, thus adaptively weighting the influence of neighbour nodes under different features. Each attention head will perform feature aggregation on the input node and its neighbour nodes. For each node i, the model first linearly transforms its eigenvectors through the learnt weight matrix *W*. Then, the features of node i and its neighbour node j will be calculated by the attention coefficient $e_{ij}$, which is calculated as follows:

(2) $$e_{ij}=\text{LeakyReLU}\left(a^{T}\left[Wh_{i}\|Wh_{j}\right]\right)$$

Here, ‖ denotes the stitching operation of features, a is the weight vector of the attention mechanism, *W* is the weight matrix, and $h_{i}$ and $h_{j}$ are the feature vectors of node i and neighbour node j, respectively.

The calculated attention coefficient $e_{ij}$ will be normalised by the softmax function to obtain the normalised attention weight $\alpha_{ij}$ of node i to its neighbour node j:

(3) $$\alpha_{ij}=\frac{\exp\;\left(e_{ij}\right)}{\sum_{k\in N\left(i\right)}\exp\;\left(e_{ik}\right)}$$

This step ensures that the sum of the attention weights of each node is 1, thus controlling the proportion of neighbour nodes contributing to the target node.

Through the normalised attention weight $\alpha_{ij}$, the neighbour node features of each node are weighted and aggregated to generate a new feature representation of node i:

(4) $$h_{i}^{\prime }=\sigma \left(\sum_{j\in N\left(i\right)}\alpha_{ij}Wh_{j}\right)$$

where σ is the activation function, we use the ELU activation function, and $h_{i}^{\prime }$ is the updated feature representation of node i. Through this process, each node not only contains its own information but also fuses the information of its neighbours.

In the multi‐head attention mechanism, each attention head independently calculates a set of attention weights and aggregated features. For each K attention head, the output features are concatenated or averaged (depending on whether it is the last layer). For the middle layer, the concatenation operation is usually used:

(5) $$h_{i}^{\prime }={\|}_{k=1}^{K}\sigma \left(\sum_{j\in N\left(i\right)}\alpha_{ij}^{k}W_{k}h_{j}\right)$$

In the last layer, the output of multi‐head attention can be averaged to avoid the growth of excessive feature dimensions. We spliced the attention scores of the two features and integrated the information through MLP to complete the fusion of multimodal data.

### 3.3 Multilayer perceptron and decoder

Multilayer perceptron (MLP) is a classical structure in neural networks, which is commonly used to extract non‐linear features from input data. It is composed of multiple fully connected layers (also called linear or dense layers), each with neurons and transformed by a non‐linear activation function. After obtaining the attention scores between nodes under each feature by GAT network calculation, we concatenate the attention score matrices of these two features to form a fused feature representation. Next, MLP takes this fused matrix as input to further integrate and extract features through several layers of non‐linear transformations. The role of MLP is to capture the high‐order relationship and complex non‐linear mapping between these multimodal features and finally help the model to complete the effective fusion of multimodal data, thereby improving the performance of downstream tasks.

The decoder is responsible for generating or reconstructing the structure (adjacency matrix) from the low‐dimensional latent space. The task of the decoder is to predict the connection relationships between cells or nodes (that is, predict the adjacency matrix) based on the feature embeddings learnt by the encoder.

(6) $$A^{\prime }=\sigma \left(Z∙Z^{T}\right)$$

Here, Z is the feature embedding matrix after MLP dimension reduction, $Z^{T}$ is the transpose of Z, and σ is the activation function (such as sigmoid) used to map the output to the range of [0,1], representing the predicted edge connection probability.

### 3.4 Loss function

In the process of optimising deep learning models, the choice of the loss function is a key factor, which directly affects its convergence speed and final prediction accuracy. To improve the accuracy of the model in the task of inferring CCIs, we employ a hybrid loss function strategy that innovatively combines the binary cross‐entropy loss with the mean squared error loss. This combination strategy can not only provide effective supervision in the reconstruction of intercellular communication network but also ensure the accurate reconstruction of multimodal features, significantly improving the training effect of the model. Formally, the proposed loss function is defined as follows:

Binary cross‐entropy loss (BCE): Used to supervise the model's reconstruction of the intercellular communication network (adjacency matrix). BCE can effectively measure the difference between the connection probability output by the model and the true label, ensuring that the model has high accuracy when inferring whether there is direct communication between cells.

(7) $${\text{BCE}}_{\left(p,q\right)}=-\frac{1}{N}\sum_{i=1}^{N}\left[q_{i}\;\log\left(p_{i}\right)+\left(1-q_{i}\right)\log\;\left(1-p_{i}\right)\right]$$

(8) $$L_{reg}=\text{norm}\times BCE\left(A^{\prime },A\right)$$

where $p_{i}$ is the ith connection probability predicted by the model. $q_{i}$ is the true label (0 or 1), indicating whether there is a connection, and N is the number of samples, and norm is the coefficient balancing positive and negative samples.

Mean squared error: To evaluate the performance of the model on feature reconstruction. We applied the mean squared error loss to gene expression features and image features, respectively, to ensure that the model could not only accurately predict cell–cell communication but also accurately reconstruct the input features reflecting the intrinsic correlations between different modal data.

(9) $${\text{MSE}}_{\left(x,\hat{x}\right)}=\frac{1}{N}\sum_{i=1}^{N}{\left(x_{i}-{\hat{x}}_{i}\right)}^{2}$$

(10) $$L_{\text{feature}}=\text{MSE}\left(\text{feature},\text{reconstruct}\_\text{feature}\right)$$

(11) $$L_{c\text{ount}}=\text{MSE}\left(\text{count},\text{reconstruct}\_\text{count}\right)$$

where $x_{i}$ is the true value of the ith feature, ${\hat{x}}_{i}$ is the ith feature reconstructed by the model, and N is the number of samples.

(12) $$L_{\text{total}}=\alpha L_{\text{reg}}+\beta L_{\text{count}}+\gamma L_{\text{feature}}$$

Here, α, β, and γ are weighting factors that weigh the reconstruction spatial network loss, the recovered image feature loss, and the recovered spatial expression matrix loss.

### 3.5 Evaluation metrics

To comprehensively evaluate the performance of the SpaGraphCCI model, we adopted several quantitative evaluation metrics, including AUC‐ROC (area under the receiver operating characteristic curve), accuracy, average precision, F1 score, and AUC‐PRC (area under the precision‐recall curve). These evaluation metrics are widely recognised in CCIs tasks and are often used to measure the predictive performance of models. In the definition of these metrics, we used TP (true positive) and FN (false negative) for cell pairs that actually had communication and those that were misclassified as having communication, FP and TN (true negative) for cell pairs that were misclassified by the model as having no communication and those that were correctly identified as having no communication. Through these evaluation criteria, we can comprehensively and objectively analyse the performance of the model in the actual cell communication identification task and provide reference for future research. The combined use of these evaluation metrics enables us to more accurately characterise the performance of the SpaGraphCCI model, ensuring its reliability in terms of accuracy, robustness, and practical applications.

AUC‐ROC (area under receiver operating characteristic curve): AUC‐ROC is an abbreviation for the area under the receiver operating characteristic curve (ROC curve). The ROC is a curve plotted with the FP rate (FPR) on the horizontal axis and true positive rate (TPR) on the vertical axis. AUC represents the area under the curve. Its definition is

(13) $$\text{TPR}=\frac{\text{TP}}{\text{TP}+\text{FN}},\text{FPR}=\frac{\text{FP}}{\text{FP}+\text{TN}}$$

(14) $$\text{AUC}\_\text{ROC}=\int_{0}^{1}\text{TPR}\left(\text{FPR}\right)d_{\text{FRP}}$$

Accuracy: Accuracy is the ratio of correctly predicted samples to the total number of samples, and the formula is

(15) $$\text{Accuracy}=\frac{TP+TN}{TP+TN+FP+FN}$$

Average precision: Average precision is an average of precision and recall based on their weighted average, which represents the model's average precision at different threshold values. The formula is as follows:

(16) $$\text{Precision}=\frac{\text{TP}}{\text{TP}+\text{FP}},\text{Recall}=\frac{\text{TP}}{\text{TP}+\text{FN}}$$

(17) $$\text{AP}\left(\text{Average}\;\text{Precision}\right)=\sum_{n=1}^{N}\left(R_{n}-R_{n-1}\right)∙P_{n}$$

Here, $P_{n}$ is the precision at the $n_{th}$ threshold, $R_{n}$ is the recall at the $n_{th}$ threshold, and n is the number of different thresholds.

F1 Score: The F1 score is the harmonic mean of precision and recall, and the formula is

(18) $$F1\;\text{Score}=2\times \frac{\text{Precision}\times \text{Recall}}{\text{Precision}+\text{Recall}}$$

AUC‐PRC (area under precision‐recall curve): AUC‐PRC is an abbreviation for the area under the precision‐recall curve (precision‐recall curve), which represents the change in precision at different recall rates. The area is obtained by integration. The formula is not simply expressed and is usually calculated numerically by numerical integration:

(19) $$\text{AUC}\_\text{PRC}=\int_{0}^{1}\text{Precision}\left(\text{Recall}\right)d_{\text{Recall}}$$

By using multiple evaluation indicators, we can comprehensively measure the performance of the SpaGraphCCI algorithm from multiple angles, ensuring that it not only performs well in correctly identifying cellular communication but also has high accuracy in correcting incorrect classifications and recovering unidentified cellular communication. By conducting quantitative analysis of these indicators, we can more accurately evaluate and compare the merits and demerits of different algorithms, thereby revealing their advantages and limitations in actual applications. These evaluation results provide scientific and objective evidence for the future clinical application of SpaGraphCCI.

## 4 EXPERIMENTS

### 4.1 Implementation details

In this paper, we implemented SpaGraphCCI using the PyTorch framework and conducted experiments on the NVIDIA GeForce RTX 3060 GPU system. The model framework used graph attention network (GAT) and was trained on 70% of the edges of each dataset. During training, we used the SGD [36] optimiser and set the initial learning rate to 1e−2 for the spot resolution‐based dataset and the Adam [37] optimiser for single‐cell resolution and set the initial learning rate to 1e−2. The maximum number of training epochs was set to 1000. We use the ReduceLROnPlateau scheduler, which adjusts the learning rate based on the validation loss. If the validation loss does not improve over 10 consecutive epochs, the learning rate is reduced by a factor of 0.05. To ensure the fairness of the experiments, all models were trained using the same data preprocessing and normalisation methods. In the best model parameter‐saving strategy, we evaluated the model's performance on the validation set after each training cycle. Specifically, if the AUC on the validation set exceeded the previously recorded best value, we would update the best value and save the current model parameters. These optimised parameters would then be loaded for evaluation on the test set. To ensure a fair comparison, we also recorded and compared the best models of existing deep learning methods (such as DeepLinc and Clarify) on the same dataset. All experimental code and detailed implementation can be accessed at the following link: https://github.com/zhanghan0307/SpaGraphCCI. In this way, we ensure the reliability and reproducibility of the experimental results, and all compared methods are tested under the same conditions, thus providing a comprehensive and impartial evaluation of the effectiveness of identifying spatial cell communication.

**FIGURE 2:**
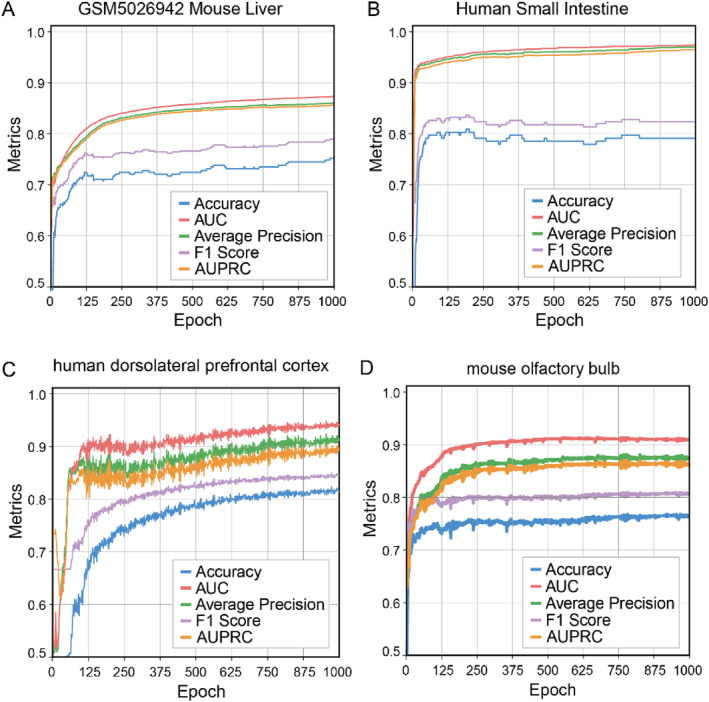
Performance of SpaGraphCCI on validation sets across four datasets with varying resolutions: (a) mouse liver, (b) human small intestine, (c) human dorsolateral prefrontal cortex, and (d) mouse olfactory bulb. Each panel illustrates the evaluation of SpaGraphCCI using five performance metrics.

As shown in Figure 2, with the increase of training times, the model performance gradually improves and tends to be stable on the validation set, indicating that our proposed SpaGraphCCI model has good convergence. Figures 2a–d show the performance across different evaluation metrics for the mouse liver (10X platform), human small intestine (10X platform), human dorsolateral prefrontal cortex (10X platform), and mouse olfactory bulb (HDST), respectively. In addition, a systematic evaluation method is used to monitor and optimise the training process of the model. After each iteration round, we closely monitor the changes of key evaluation metrics to ensure the best balance between the learning ability and generalisation ability of the model. In this way, we can obtain fully optimised model parameters at the end of training, which guarantees that the model has the best performance on both the validation set and the test set, and the results on the test set are shown in Table 1. This strategy not only significantly improves the prediction accuracy of the model but also enhances its robustness and effectiveness in practical applications. Experimental results show that SpaGraphCCI not only has strong convergence characteristics but also shows good generalisation ability in complex biological data scenarios, which shows a wide range of application potential in ST cell communication recognition tasks. We have shown in Supplementary Table 1 the running time and average memory usage of SpaGraphCCI for 1000 epochs of training when inferring cell–cell communication under different datasets.

**TABLE 1:** Performance of SpaGraphCCI on the validation set.

Dataset	AUC	ACC	F1	PRC	AP
GSM5026942 mouse liver	0.880	0.786	0.812	0.829	0.832
Human small intestine	0.951	0.768	0.806	0.965	0.965
Human breast cancer	0.860	0.729	0.772	0.853	0.853
Mouse olfactory bulb	0.907	0.762	0.804	0.872	0.872
GSM5026931 mouse liver	0.880	0.743	0.786	0.874	0.874
Human colon	0.965	0.763	0.807	0.969	0.969
Human dorsolateral prefrontal cortex	0.937	0.822	0.849	0.905	0.905

### 4.2 Comparative experiments

**FIGURE 3:**
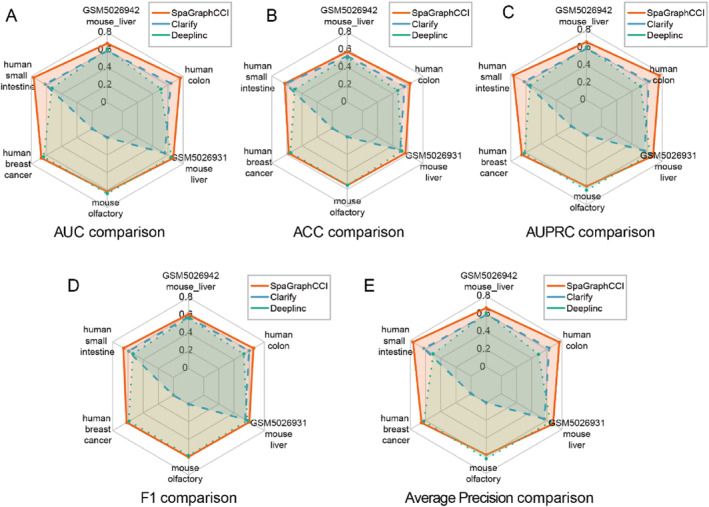
Comparison of SpaGraphCCI, DeepLinc, and Clarify across six datasets with varying resolutions, evaluated using five performance metrics: (a) AUC, (b) ACC, (c) AUPRC, (d) F1 score, and (e) Average Precision. Each panel highlights the performance differences among the three methods on the respective metric.

To objectively verify the effectiveness of our method, we compared SpaGraphCCI with other deep learning algorithms, namely DeepLinc and Clarify, which use the same approach for constructing cell graphs and are able to infer cell–cell communication at single‐cell resolution. To ensure fairness, we ran these methods on the same datasets and with default parameters. The radar chart in Figure 3 shows the performance differences among the methods.

We evaluated the performance of the three methods on six different datasets including two species and different resolutions. The edges in the initial adjacency graph A were marked as positive samples and randomly sampled 1:1 positive and negative edges for subsequent comparisons in both the training and test sets. To evaluate the reconstruction performance, we calculated the average AUROC (Figure 3a), accuracy (Figure 3b), AUPRC (Figure 3c), F1 score (Figure 3d) and average precision (Figure 3e) values for reconstructing the test set edges during training. The results show that SpaGraphCCI achieved the best performance on five datasets; it was slightly inferior to DeepLinc on the HDST platform mouse olfactory bulb dataset, which may be due to the sparse data. Clarify was unable to successfully reconstruct cell–cell communication in the HDST platform human breast cancer and mouse olfactory bulb datasets. Detailed AUC comparisons are provided in Table 2.

**TABLE 2:** Comparison of AUC between SpaGraphCCI and DeepLinc, clarify.

	GSM5026942_mouse_liver	human_small_intestine	human_breast_cancer	mouse_olfactory	GSM5026931_mouse_liver	human_colon
**DeepLinc**	0.765	0.659	0.793	**0.801**	0.801	0.634
**Clarify**	0.773	NA	NA	0.698	0.799	0.773
**SpaGraphCCI**	**0.844**	**0.963**	**0.835**	0.774	**0.838**	**0.957**

*Note*: The bold values indicate highest scoring.

### 4.3 Stability experiments

**FIGURE 4:**
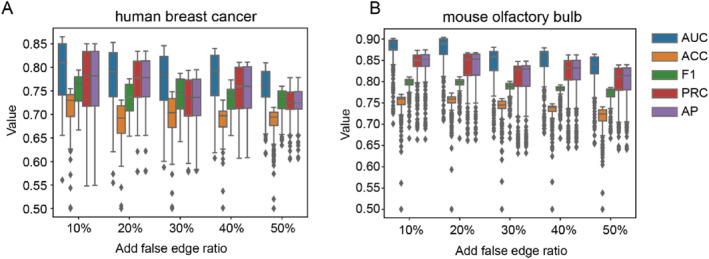
Stability test of SpaGraphCCI under varying noise levels. Performance on the validation set is evaluated by randomly adding 10%–50% false edges to the training set edge: (a) human breast cancer dataset and (b) mouse olfactory bulb dataset.

To verify the robustness (i.e. noise tolerance) of SpaGraphCCI, we intentionally added randomly generated false edges to the interaction network of the training set, where the edges were randomly selected to be false interactions between cell pairs and labelled as 0. We used this high‐noise data as input and conducted experiments on the HDST human breast cancer dataset and mouse olfactory bulb dataset. The results are shown in Figure 4a,b where SpaGraphCCI can effectively distinguish between the false edges that were artificially added and the predefined true edges, even when the number of artificially added false edges reached half of the original number of edges. SpaGraphCCI can accurately filter out a large number of noisy edges and recover the true CCIs with high precision (Figure 4a,b). These results show that SpaGraphCCI has a high tolerance for random noise interactions and exhibits superior noise‐tolerant performance.

### 4.4 Ablation study

**FIGURE 5:**
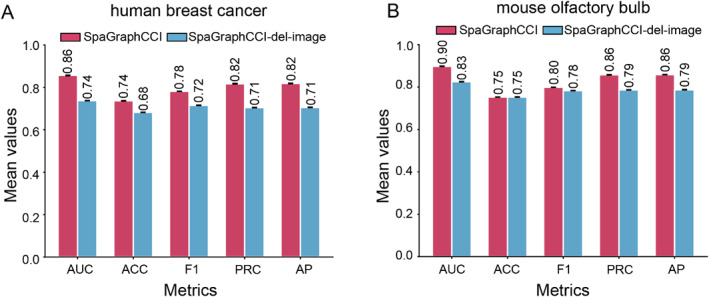
Performance comparison of SpaGraphCCI with and without image features. Ablation experiments are conducted on (a) the human breast cancer dataset and (b) the mouse olfactory bulb dataset.

In this study, we deeply explore the key role of image features in enhancing model performance. We used five evaluation metrics, including AUC, ACC, F1, PRC and average precision, to perform ablation experiments on two datasets of human breast cancer and the mouse olfactory bulb. Each dataset was divided into training set, validation set and test set. The average scores of the five metrics for human breast cancer and the mouse olfactory bulb in the validation set are shown in Figure 5a,b, and the results for the test set are shown in Table 3. The experimental results show that the performance of the model is significantly reduced after removing image features, for example, the AUC on the human breast cancer dataset is reduced by 12% (Figure 5a). The experimental result indicates that image features play an important role in cell communication inference and shows the great potential of multimodal fusion in improving the performance of the model.

**TABLE 3:** Influence of image features on model performance on the test set.

	Human breast cancer		Mouse olfactory bulb	
	SpaGraphCCI	SpaGraphCCI‐del‐image	SpaGraphCCI	SpaGraphCCI‐del‐image
AUC	0.860	0.715	0.907	0.823
ACC	0.729	0.667	0.762	0.738
F1	0.772	0.692	0.805	0.771
PRC	0.853	0.678	0.872	0.786
AP	0.853	0.679	0.872	0.787

### 4.5 SpaGraphCCI is able to predict distal cell–cell communication in human breast cancer

**FIGURE 6:**
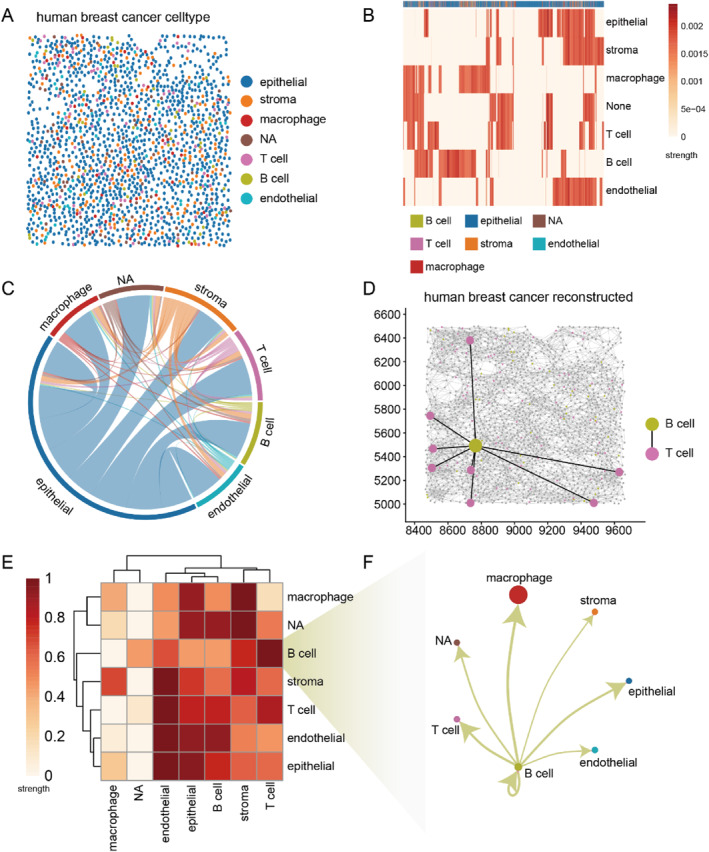
SpaGraphCCI is able to infer distal cell communication for the human breast cancer dataset. (a) Cell types of cells in human breast cancer. (b) The strength of communication between individual cells and between cell types. (c) The amount of communication between cell types. (d) SpaGraphCCI can infer distal communication. (e) The strength of communication between cell types. (f) The amount of communication between B cells and other cell types.

In this case study, we applied SpaGraphCCI to the spatial transcriptome data of human breast cancer. Annotation results show that the dataset contains 1765 cells and 7 cell types. The spatial location of the cells and their cell types were plotted (Figure 6a). To analyse these interactions, we calculated the communication strength between individual cells and cell types and visualised it with a heatmap (Figure 6b). Communication strength was defined as the proportion of communication edges between a single cell and a specific cell type out of all communication edges of that cell type. Additionally, we visualised the proportion of cells and the number of communications between different cell types in the dataset using a circle plot in Figure 6c. The epithelial cells were significantly more numerous than other cell types, and they had interactions with multiple cell types. Studies showed that T cells and B cells interact through the inducible costimulatory molecule ICOS and its receptor ICOSL to promote complex immune interactions [38, 39, 40]. The advantage of SpaGraphCCI is its ability not only to infer communication between cell types but also to reveal distal interactions between cells at single‐cell resolution. Through our analyses, we have identified distal communication between T cells and B cells (Figure 6d). This phenomenon may be due to the dependence of T cells and B cells on cytokine‐mediated long‐distance communication [24]. To further research the communication strength between cell types, we used a heatmap (Figure 6e) to visualise the distribution of overall communication strength. When analysing the interactions between B cells as the sending cells and other cell types as receiving cells, we found that the communication between B cells and macrophages was significantly stronger than that with other cell types (Figure 6f).

## 5 DISCUSSION

With the rapid development of spatial transcriptomic technologies, researchers are able to deeply dissect highly heterogeneous cellular organisations in both physiological and pathological contexts. This provides unprecedented opportunities to understand the complex interactions between cells. However, it remains a challenging problem to comprehensively and accurately infer CCIs in spatial transcriptome profiles at different resolutions. Firstly, spatial transcriptome atlases are only random snapshots from a limited number of tissue sections, and the interaction information embedded in cellular organisation patterns may only represent local features of the entire cellular landscape. Second, current spatial transcriptome data still face many technical limitations, such as bias, insufficient gene coverage, low signal‐to‐noise ratios, as well as data sparsity and batch effects due to high‐throughput technologies [9, 41, 42, 43]. These problems require researchers to develop new methods that are highly robust to technical noise and data missing.

SpaGraphCCI can effectively eliminate noise and accurately infer distal CCIs by integrating multimodal features and deeply mining spatial transcriptome data. SpaGraphCCI does not rely on prior information about ligand–receptor pairs but assumes that information about CCIs is already encoded in the spatial transcriptome. By learning the observed cell interactions, SpaGraphCCI was able to effectively train the model and infer missing interactions. SpaGraphCCI shows significant advantages in identifying cellular communication in the spatial transcriptome, especially in inferencing distal CCIs, which can accurately capture intercellular communication signals. In addition, SpaGraphCCI has excellent robustness, which can stabilise the trained model and obtain reliable results even when half of the FP samples are included in the data.

Overall, in the face of high noise data, SpaGraphCCI can fully mine the characteristics of multimodal data, effectively remove noise, and accurately identify CCIs, which reflect excellent anti‐noise ability and provide strong support for cell communication research.

## 6 CONCLUSION

SpaGraphCCI is an innovative computational method that aims to reconstruct CCI networks from ST data using deep learning strategies. The method integrates ST gene expression profiles and image features to capture potential patterns, filter out noise, and efficiently infer both distant and proximal CCIs. Compared to existing methods, SpaGraphCCI exhibits significant advantages in handling highly sparse and noisy ST data, especially in overcoming technical limitations and recovering hidden CCIs. SpaGraphCCI provides a helpful tool for analysing ST data, showcasing the potential of deep learning in large‐scale, biologically complex datasets.

## AUTHOR CONTRIBUTIONS

**Han Zhang**: Data curation; formal analysis; methodology; validation; writing–original draft. **Ting Cui**: Data curation; formal analysis. **Xiaoqiang Xu**: Data curation; formal analysis. **Guangyu Sui**: Formal analysis. **Qiaoli Fang**: Formal analysis. **Guanghao Yang**: Formal analysis. **Yizhen Gong**: Data curation. **Sanqiao Yang**: Formal analysis. **Yufei Lv**: Data curation; writing–review & editing. **Desi Shang**: Writing–review & editing.

## CONFLICT OF INTEREST STATEMENT

The authors declare no conflicts of interest.

## Supporting information

Supplementary Material

## Data Availability

The datasets of the mouse olfactory bulb and human breast cancer were downloaded from the data portal at https://portals.broadinstitute.org/single_cell/study/SCP420. The dataset of the human dorsolateral prefrontal cortex was downloaded from the datasets at https://bio.liclab.net/spatialref/. The datasets of the human colon and human small intestine were downloaded from GEO under accession number GSE165141. Two datasets of the mouse liver were downloaded from GEO under accession number GSE116222. Our code, and experimental results are available at https://github.com/zhanghan0307/SpaGraphCCI.
