# Supplementary material for: SpaGraphCCI: Spatial cell–cell communication inference through GAT‐based co‐convolutional feature integration
Source: IET Syst Biol. 2025 Jan 23;19(1):e70000. doi: 10.1049/syb2.70000 (PMC11771809; doi:10.1049/syb2.70000)
Supplement: Supplementary file 1 — Supplementary Material [file SYB2-19-e70000-s001.docx]

**Supplementary Table 1** The size of the dataset and the running time of 1000 epochs and the average memory footprint.

| **Dataset** | **Spot/cell number** | **Gene number** | **Time (running 1000 epochs)** | **Memory (every epoch)** |
| --- | --- | --- | --- | --- |
| **Mouse brain bulb** | 1981 | 1981 | 9155.5s | 7819.09MB |
| **Human breast cancer** | 1765 | 1765 | 8540.8s | 7391.12MB |
| **Human small intestine** | 346 | 13231 | 1872.5s | 3997.09MB |
| **Human colon** | 1080 | 13298 | 4865.6s | 5441.85MB |
| **GSM50526942 mouse liver** | 590 | 9655 | 2797.5s | 4380.22MB |
| **GSM5026931 mouse liver** | 684 | 9655 | 3023.9s | 4560.30MB |
| **Human dorsolateral prefrontal cortex** | 4226 | 14186 | 21119.6s | 11754.84MB |
